# Phytochemical profiling of antimicrobial and potential antioxidant plant: *Nepeta cataria*

**DOI:** 10.3389/fpls.2022.969316

**Published:** 2022-09-26

**Authors:** Ali Nadeem, Hira Shahzad, Bashir Ahmed, Tudor Muntean, Maaz Waseem, Aisha Tabassum

**Affiliations:** ^1^Plant Pathology Lab, Department of Biological Sciences, International Islamic University, Islamabad, Pakistan; ^2^Department of Plant Biology, Stockbridge School of Agriculture, University of Massachusetts, Amherst, MA, United States; ^3^International Centre for Public Health (ICPH), New Jersey Medical School, Rutgers, The State University of New Jersey, New Brunswick, NJ, United States; ^4^Clinical Epigenetics Lab, University Institute of Biochemistry and Biotechnology, PMAS Arid Agriculture University, Rawalpindi, Pakistan; ^5^Atta-Ur-Rahman School of Applied Biosciences (ASAB), National University of Sciences and Technology (NUST), Islamabad, Pakistan; ^6^Department of Biochemistry, University of Sialkot, Sialkot, Pakistan

**Keywords:** *Nepeta cataria*, gas chromatograph/mass spectrometry (GC/MS), antibacterial susceptibility testing (AST), antioxidants, phytochemicals

## Abstract

Traditional and phytochemical studies have confirmed the richness and diversity of medicinal plants such as *Nepeta cataria* (*N. cataria*), but more studies are needed to complete its metabolite profiling. The objective of this research was to enhance the metabolomic picture and bioactivity of *N. cataria* for better evaluation. Phytochemical analysis was performed by bio-guided protocols and gas chromatography-mass spectrometry (GC/MS). For this, solvents such as methanol, ethanol, water, acetone, and hexane were used to extract a wide number of chemicals. Antibacterial analysis was performed using the 96-well plate test, Kirby Bauer's disk diffusion method, and the resazurin microdilution test. Antioxidant activity was determined by the DPPH assay and radical scavenging capacity was evaluated by the oxygen radical absorbance capacity (ORAC) assay. GC/MS analysis revealed a total of 247 identified and 127 novel metabolites from all extracts of *N. cataria*. Water and acetone extracts had the highest identified metabolites (*n* = 79), whereas methanol extract was the highest in unidentified metabolites (*n* = 48). The most abundant phytochemicals in methanol extract were 1-isopropylcyclohex-1-ene (concentration = 27.376) and bicyclo [2.2.1] heptan-2-one (concentration = 20.437), whereas in ethanol extract, it was 9,12,15-octadecatrienoic acid (concentration = 27.308) and 1-isopropylcyclohex-1-ene (concentration = 25.854). An abundance of 2 methyl indoles, conhydrin, and coumarin was found in water extracts; a good concentration of eucalyptol was found in acetone extract; and 7,9-di-tert-butyl-1-oxaspiro is the most abundant phytochemicals in hexane extracts. The highest concentration of flavonoids and phenols were identified in hexane and methanol extracts, respectively. The highest antioxidant potential (DPPH assay) was observed in acetone extract. The ethanolic extract exhibited a two-fold higher ORAC than the methanol extract. This examination demonstrated the inhibitory effect against a set of microbes and the presence of polar and non-polar constituents of *N. cataria*. The results of this study provide a safe resource for the development of food, agriculture, pharmaceutical, and other industrial products upon further research validation.

## Introduction

The *Nepeta* genus belongs to the family Lamiaceae, which is rich in bioactive secondary metabolites. The word Cataria was derived from the Latin word for cat, “Cathus.” *N. cataria* is a perennial herb that grows to a height of 50–100 cm (Scott, 2003). It has been found predominately in the regions of southern and eastern Europe, the Middle East, Central Asia, and China. Bioactive compounds of *N. cataria* have prehistorically been used and have a wide range of biological activities, including analgesic, anti-asthmatic, anti-cancer, anti-inflammatory, and antimicrobial properties. *Nepeta cataria* essential oil and metabolites have important applications in the pharmaceutical, agrochemical, and food industries (Sharma et al., 2021). Researchers found them to be antifungal, antibacterial (Bandh and Kamili, 2011; Sharma et al., 2019), antioxidant (Adiguzel et al., 2009), insecticidal, anti-inflammatory, anti-nociceptive, and potentially spasmolytic (Pargaien et al., 2020; Giarratana et al., 2017). Essential oils, flavonoids, phenolic acid, steroids, terpenoids, and terpenoid hydrocarbons have all been found in this plant.

*Nepeta cataria* has widely been used to treat diarrhea because of spasmolytic and myorelaxant metabolites in its extracts (Gilani et al., 2009). Essential oils of *N. cataria* have a promising impact on raw materials of industrial food importance (Frolova et al., 2020). Studies established the presence of nepetalactones in catnip essential oil by TLC and GC–MS analysis. Using GC/MS analysis, three populations of *N. crassifolia* and four populations of *N. nuda* were studied (Sharma et al., 2021).

Essential oils and flavonoids have typically been linked to the therapeutic benefits of *Nepeta* species. Prior investigations on the essential oils of *N. cataria* identified nepetalactone as a major constituent (Mamadalieva et al., 2017; Sharma et al., 2019). In a recent study, water-based extracts of *N. cataria* significantly inhibited herpes virus replication in humans (Hinkov et al., 2020). Previously, *N. cataria* has been used to alleviate symptoms of bronchial asthma, bronchitis, and bronchial congestion. The traditional herbal medicine derived from these along with other medicinal plants may have multiple applications, including symptom relief for people with COVID-19 and the development of effective antiviral medicines. During the severe acute respiratory syndrome coronavirus (SARS-CoV-2) pandemic, also termed COVID-19, leaves of *N. cataria* were used to alleviate symptoms of the disease (Khan et al., 2021). Essential oils from *Nepeta* species that naturally produce nepetalactones can be synthesized in other regions and then be distilled to serve as a natural source of efficient *Aedes aegypti* repellent for effective dengue prevention (Reichert et al., 2019). Previous studies demonstrate that *N. cataria* essential oils effectively reduced liver damage caused by acetaminophen and enhanced mRNA expression of uridine diphosphate glucuronosyltransferases (UGTs) and sulfotransferases (SULTs) and decreased CYP2E1 activity (Tan et al., 2019). It has been shown that *N. cataria* and its derivatives have been used to treat gastrointestinal and respiratory disorders. They have also been reported for their effective antibacterial, antiviral, and antioxidant activities (Sharma et al., 2019). Porcine reproductive and respiratory syndrome virus (PPSRV) affects pigs and causes reproductive failure in developing pigs. According to the findings of a study, the load of PRRSV could be greatly reduced by using *N. cataria* hydrosol. It is strongly recommended that further research be conducted into the antiviral processes and characteristics of these plant hydrosols, both *in vitro* and *in vivo* (Kaewprom et al., 2017).

Recent research has been focused on the essential oils and antibacterial properties of plants, as they have been utilized to increase the shelf life of foods and in traditional medicine (Ergün, 2021; Özkan et al., 2021). Numerous studies demonstrate that the antibacterial and antifungal properties of *N. cataria* are mostly attributable to the essential oil constituents. Surprisingly, less is known about the antimicrobial activity of catnip essential oil. In these investigations, the antimicrobial activity of catnip essential oil was investigated on a limited number of bacteria or fungi (Angelini et al., 2006; Suschke et al., 2007; Bourrel et al., 2011).

In the past two decades, the antioxidant effect of the essential oils and/or extracts of medicinal and aromatic plants has received considerable attention. Therefore, these extracts can be employed as safe and effective synthetic preservative replacements. Natural antioxidants have been investigated extensively for their ability to protect organisms and cells against oxidative stress-induced damage, which is believed to be a cause of aging, degenerative illnesses, and cancer (Sharma et al., 2019). It has been known for some time that plant extracts and/or essential oils possess antioxidant properties. However, less is known about the antioxidant activity of the essential oil or extract of *N. cataria*.

In another study, aromatic and medicinal plants from Turkey have been characterized and reported on the antibacterial and antioxidant activities of *N. cataria*'s essential oil, methanol extract, and its essential oil composition. They also highlighted essential oil to contain 4aβ, 7α, 7aβ-nepetalactone, 4aα, 7α, 7aβ-nepetalactone, 1,8-cineole, and elemol as major oil constituents in *N. crassifolia* (Dabiri and Sefidkon, 2003), while 7aβ-nepetalactone, 4aα, 7α, 7aβ-nepetalactone, pulegone, and piperitenone oxide were identified in *N. nuda* (Narimani et al., 2017). Research studies focused mainly on essential oil extracts of *N. cataria*, which indicated a need to study its metabolites in polar and nonpolar solvents. Our team was motivated to explore the constituents of *N. cataria*, based on polarity, *via* minor adjustments to already established lab protocols.

## Materials and methods (experimental)

### Plant collection

*Nepeta cataria* was collected from Swat (Himalayas), Khyber Pakhtunkhwa, Pakistan (35°22′59.99″ N, 72°10′60.00″E), locally named as catnip mint/catmint (in northern Pakistan) and Badranj boya (in central Pakistan). Species verification and identification were done at the National Herbarium, and they confirmed and identified it as *N. cataria*. Furthermore, it was cleaned, rinsed, dried, and preserved at the Antimicrobial Biological Laboratory (AMBL), International Islamic University Islamabad, Islamabad, Pakistan.

### Plant extraction and filtration

*Nepeta cataria's* stem and leaves were rinsed, dried, and grounded in a fine powder by a lab grinder carefully. Fine powder was soaked separately in methanol, ethanol, water, acetone, and hexane using 1:10 ratio for 24–48 h at room temperature, to increase the maximum solubility. Filtrations and extraction were done using Whatman's # 41 and rota-evaporator at Stockbridge Medicinal and Aromatic Lab, University of Massachusetts Amherst, USA. Extracts were labeled and aliquoted in glass vials at 4°C until further use.

### Phytochemical analysis

#### Qualitative analysis

Saponins and phenolic compounds, water-soluble and insoluble phenols, alkaloid flavonoids, poly-steroids, terpenoids, cardiac glycosides, free and combined anthraquinones, tannins, and alkaloids were chemically identified in all plant extracts (Prabhavathi et al., 2016).

#### Quantitative analysis—Phenols and flavonoids

Concentrations of phenols and flavonoids were identified in all extracts of *N. cataria via* established protocols previously explained in Nadeem et al. (2021).

#### GC/MS analysis of *N. cataria* extracts

The GC/MS is the widely adopted technique for the detection of biologically active compounds, i.e., metabolites. A set of extracts, methanol, ethanol, water, acetone, and hexane were subjected to GC/MS analysis to detect bioactive phytochemicals. Phytochemical compounds were identified and presented with their compound names, molecular formulas, molecular weight, and retention times (RT) using NIST Library 17.

Metabolic profiling of *N. cataria* extracts was conducted *via* GC/MS (Bruker Scion 456 GC, EVOQ triple quadrupole GC-MS/MS). A column of 15 m was used with a diameter and film thickness of 0.25 mm. The flow rate of helium as a carrier gas was 1.5 ml/min. For gas chromatography, temperature conditions were 45°C for 3 min, 250°C at 8°C/min for 10 min. Injection volume was 1 μl [varying split ratio (5:1/15:1/20:1), range (45–350 m/z)]. Automated Mass Spectral Deconvolution and Identification System (AMDIS) Software MSWS 8 for GC/MS and NIST library were used for compilation of all results.

### Antibacterial activity

Bacterial cultures (Table 1) were grown on a tryptic soy broth (TSB) medium (Thermo Fisher Scientific, USA) (Nadeem et al., 2021). To evaluate antibacterial susceptibility testing (AST) of *N. cataria* extracts, three different methods were used, i.e., 96-well test, Kirby-Bauer disk diffusion, and resazurin-based well plate microdilution method.

**Table 1:** Microbial profile of bacterial ingredients used in the antimicrobial analysis.

**Microorganism**	**Accession number**	**Strain**
*Escherichia coli*	ATCC_25922	Gram negative
*Klebsiella oxytoca*	ATCC_43863	
*Salmonella enterica*	ATCC_14028	
*Shigella sonnei*	ATCC_25931	
*Citrobacter ferundii*	ATCC_8090	
*Bacillus subtilis*	ATCC_6051	Gram positive
*Lactococcus lactis*	ATCC_LMO230	
*Listeria monocytogenes*	ATCC_LM21	
*Micrococcus luteus*	ATCC_4698	
*staphylococcus aureus*	ATCC_25923	

#### The 96-well plate method

In each well of a 96-well microtiter plate, 100 μl of plant extract and TSB media were used. Each plant extract was checked at five bacterial concentrations (i.e., 1,000, 500, 250, 125, and 62.5 μg) for optimum antimicrobial potential. Only TSB medium was added to negative control well to ensure sterility of media. A single negative control lacked plant extract to observe normal bacterial growth. Microtiter plates were incubated for 24 h before reading at 570 nm. Chloramphenicol as standard was used to evaluate the results. Bacterial inhibition was calculated *via* the following formula:


$$\begin{array}{l} Bacterial\;inhibition=\frac{\text{OD in control}-\text{OD in treatment}}{\text{OD in control}}\times 100 \end{array}$$

#### Kirby-Bauer disk diffusion method

Solidified agar plates were used to analyze the antimicrobial potential of *N. cataria* extracts. Paper disks of 10 mm were soaked in 20 μl extracts, then placed on prepared culture plates and incubated for 24 h at a 25–35°C temperature. Paper disks (10 mm) were soaked in 20 μl of distilled water as a negative control to avoid any influence on bacterial growth (Sarin and Bafna, 2012). Aseptic conditions were maintained *via* working in a laminar flow. All extracts were tested in biological triplicates, and results were represented as average values of inhibition zones in mm ± standard deviation.

#### Resazurin-based well plate microdilution method

Resazurin solution was prepared (121.5 mg resazurin powder in 18 ml of ddH_2_O) and mixed for 1 h (pH = 7.4). TSB liquid medium and *N. cataria* extracts (100 μl each) were added to each well. Plant extract was added in serial dilution to separate wells. Each well was supplied with 106 CFU/ml of bacterial inoculum. Double negative control well was supplied with TSB media only. Single negative control well was supplied with TSB media and bacterial culture. Plates were incubated overnight and then 20 μl of resazurin was added to each well and incubated for another 4 h. Absorbance at 550–590 nm was read *via* spectrophotometer (SPECTRA MAX M2e plate reader) (Packialakshmi and Naziya, 2014).

### DPPH antioxidant assay

The Bersuder (Edewor and Usman, 2011) method was used for antioxidant determination *via* DPPH radical scavenging assay. All solvent extracts were mixed with DMSO addition and DPPH-ethanol reagent was made separately. Plant-DMSO mix was saturated with DPPH-ethanol reagent for 6 h. Negative control was prepared by dissolving ascorbic acid in DMSO (50–500 μmol/L), which was used to generate calibration curve with 517 nm absorbance read *via* SPECTRA MAX M2e plate reader (Packialakshmi and Naziya, 2014).

### Oxygen radical absorbance capacity assay protocol

Various dilutions of methanolic and extracted samples were mixed with buffered saline (10 mM, pH 7.6). Decaying of fluorescein induced by AAPH was compared to Trolox (positive control) over 120 min to evaluate the antioxidant activity *via* the SPECTRAMAX M2e Plate reader. Results were presented as μM Trolox Equivalent/100 μl of plant extract.

### Statistical analysis

The results of all the experiments were analyzed under a complete randomized design (CRD) with three replications for each treatment. Results were statistically analyzed using GraphPad Prism and Microsoft Office Excel 2016 version. Means were calculated, and one-way analysis of variance (ANOVA) test was performed for multiple comparisons of all the mean values. Mean differences were calculated by least significant difference (LSD) at 0.05 probability.

## Results

*Nepeta cataria* contains medicinally important phytochemicals along with many unknown metabolites that need further studies (Elshikh et al., 2016; Mir et al., 2016). High antioxidant activity was exhibited in acetone extract of *N. cataria*. Moreover, high flavonoid content was found in water and hexane extracts, and methanol extracts were specifically rich in phenols.

### Preliminary phytochemical analysis

#### Qualitative phytochemical analysis of *N. cataria*

**Figure 1:**
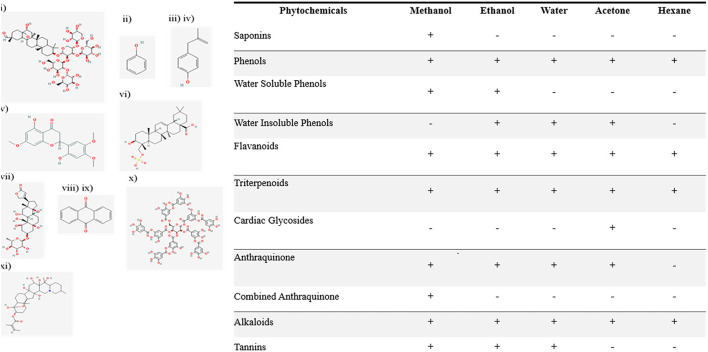
Qualitative analysis of phytochemicals in polar and non-polar extracts of *Nepeta cataria*. List of phytochemicals from (i) to (xi) were identified in various polar and non-polar extracts. The 2-D structure of phytochemicals are supported *via* PubChem.

Saponins were found in the methanol-based extracts of *N. cataria*. Phenols were positive in all extracts and showed high μg/ml concentration in methanol. Water-soluble phenols were present in all the polar solvents only. Water insoluble phenols were identified in the ethanol, acetone, and hexane-based extracts. A qualitative test for flavonoids was carried out, and the development of intense yellow color indicates presence of flavonoids (Figure 1). A qualitative test for terpenoids was conducted by observing a reddish-brown coloration development, which confirms the positive test results in all extracts. Cardiac glycosides were indicated *via* development of green-blue color. Acetone-based extracts were positive only. Free anthraquinones were present in all extracts of *N. cataria* except hexane-based extract. Combined anthraquinones were only present in methanol-based extract of *N. cataria*. Qualitative tests for tannins were found positive only in extraction of polar solvents. Alkaloids were present in all the extracts of *N. cataria*.

### DPPH antioxidant activity

**Figure 2:**
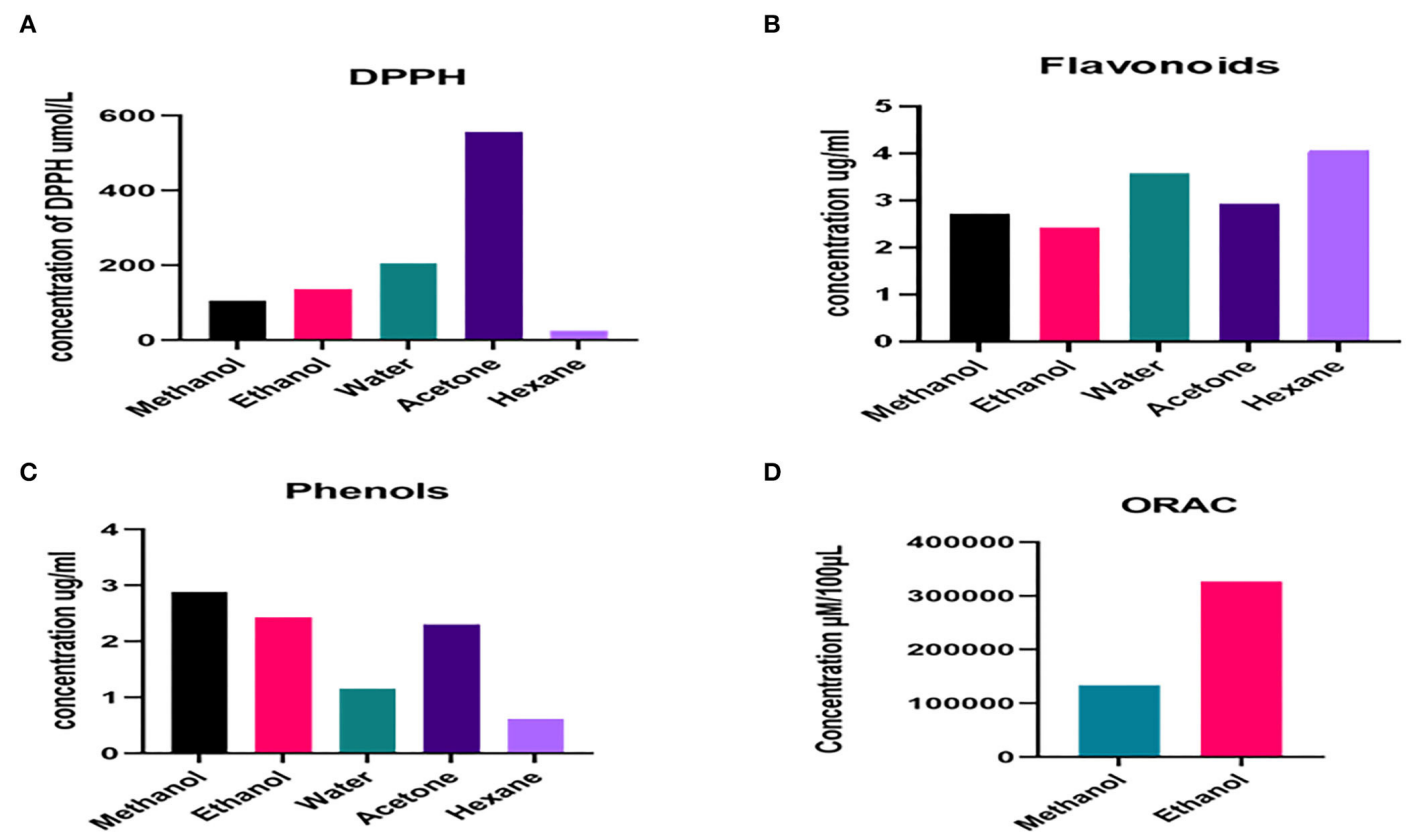
Quantitative analysis of phytochemicals **(A)** DPPH mediated antioxidant activity, **(B)** flavonoids concentration, **(C)** phenols concentration, **(D)** oxygen radical absorbance capacity values.

presence of antioxidants was determined in *N. cataria* extracts in a set of different extractions and was measured spectrophotometrically, results were drawn as μmol of ascorbic acid equivalents/L, and the results are given in Figure 2A. The presence of antioxidants was found in the following order: acetone extracts > water extracts> ethanol extracts > methanol extracts > hexane extracts.

### Total flavonoid and phenol content

The flavonoids in polar and non-polar extracts of *N. cataria* were quantified in terms of μg of catechin equivalents/ml. Hexane and water-based extracts showed high levels of flavonoids as compared to acetone, methanol, and ethanol-based extracts. Flavonoid results are summarized in Figure 2B. Several other studies prove the presence of flavonoids in *N. cataria* extract and indicate therapeutic potential for lung cancer because of its flavonoid content (Naguib et al., 2012; Yang et al., 2020).

The methanol, ethanol, water, acetone, and hexane extracts of *N. cataria* were examined in terms of μg of gallic acid equivalents per ml to quantify levels of total phenols. Methanol, acetone, and ethanol-based extracts showed the maximum presence of phenols as compared to water and hexane-based extracts. The order of phenolics (Figure 2C) presence in the sample was found as follows:


$$\begin{array}{l} \text{Methanol}\;\text{extracts}>\text{Ethanol}\;\text{extracts}>\text{Acetone}\;\text{extracts}\\ \;>\text{Water}\;\text{extracts}>\text{Hexane}\;\text{extracts}. \end{array}$$

### ORAC assay on *N. cataria* extracts

Oxygen radical absorbance capacity was performed to study the antiradical activity in methanol and ethanol extract of *N. cataria*. Results showed two-fold higher ORAC in ethanolic extracts than methanol extract (Figure 2D), signifying our results of DPPH, free radical scavenging activity (Lucas-Abellán et al., 2008).

### Determination of antibacterial activity

#### Percentage growth inhibition by 96-well method

**Figure 3:**
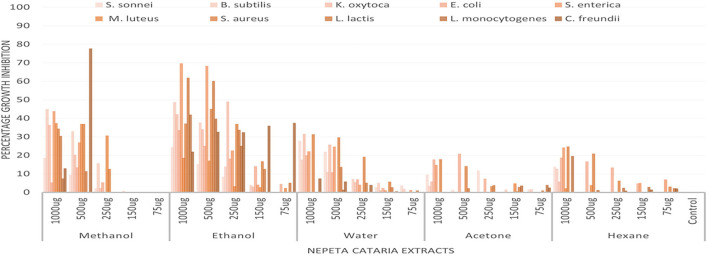
Percentage growth inhibition of bacterial strains by *Nepeta cataria* plant extracts in different solvents at different dose levels (96 well method).

Percentage growth inhibition of each tested bacteria, *viz., Shigella sonnei, Bacillus subtilis, Klebsiella oxytoca, Escherichia coli, Salmonella enterica, Micrococcus luteus, and Staphylococcus aureus (S. Lactococcus lactis, Listeria monocytogenes*, and *Citrobacter freundii)*. Percentage growth inhibition of bacterial isolates is given in Figure 3.

#### Kirby-Bauer disk diffusion method

Kirby disk diffusion method was followed to measure the antimicrobial efficacy of plant extracts by the zone of inhibition (mm) *in vitro* conditions on solidifying agar media. Chloramphenicol was used as a standard and zone of inhibition was >25 mm for all strains according to CLSI guidelines (Humphries et al., 2018).

#### Resazurin-based well plate microdilution method

**Figure 4:**
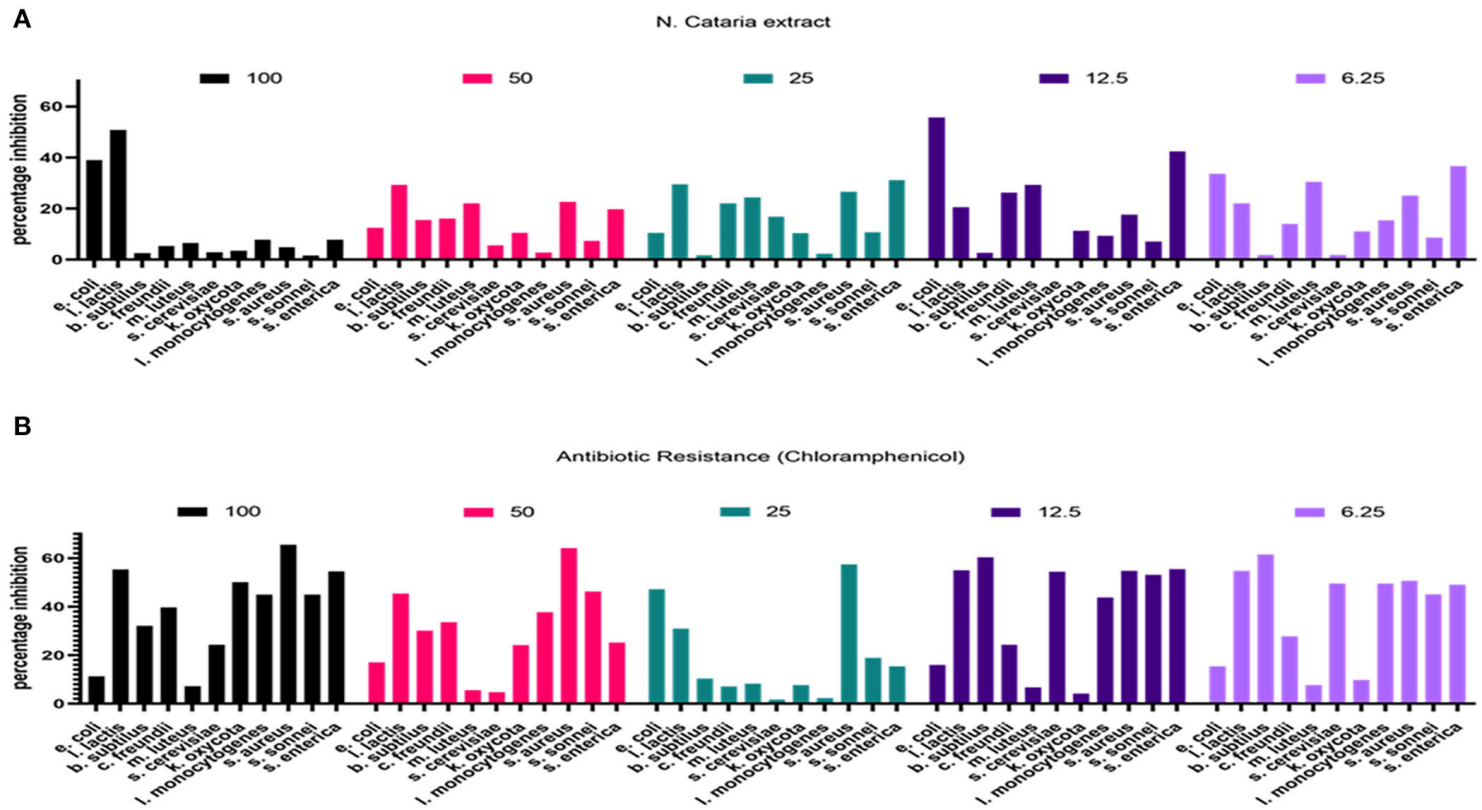
**(A,B)** Percentage inhibition of bacterial growth determined in comparison with the growth inhibition by chloramphenicol (resazurin method).

The resazurin method was used to check the antimicrobial efficacy of each prepared plant extract against tested bacterial agents. Chloramphenicol was used as a positive control at 6.25–100 μl/ml dose levels, and data on percentage bacterial growth inhibition was recorded. Plant extract of *N. cataria* showed a varied efficacy against all the tested bacterial isolates compared to the positive and negative control, and results are presented in Figure 4.

### GC/MS analysis of *N. cataria*

**Figure 5:**
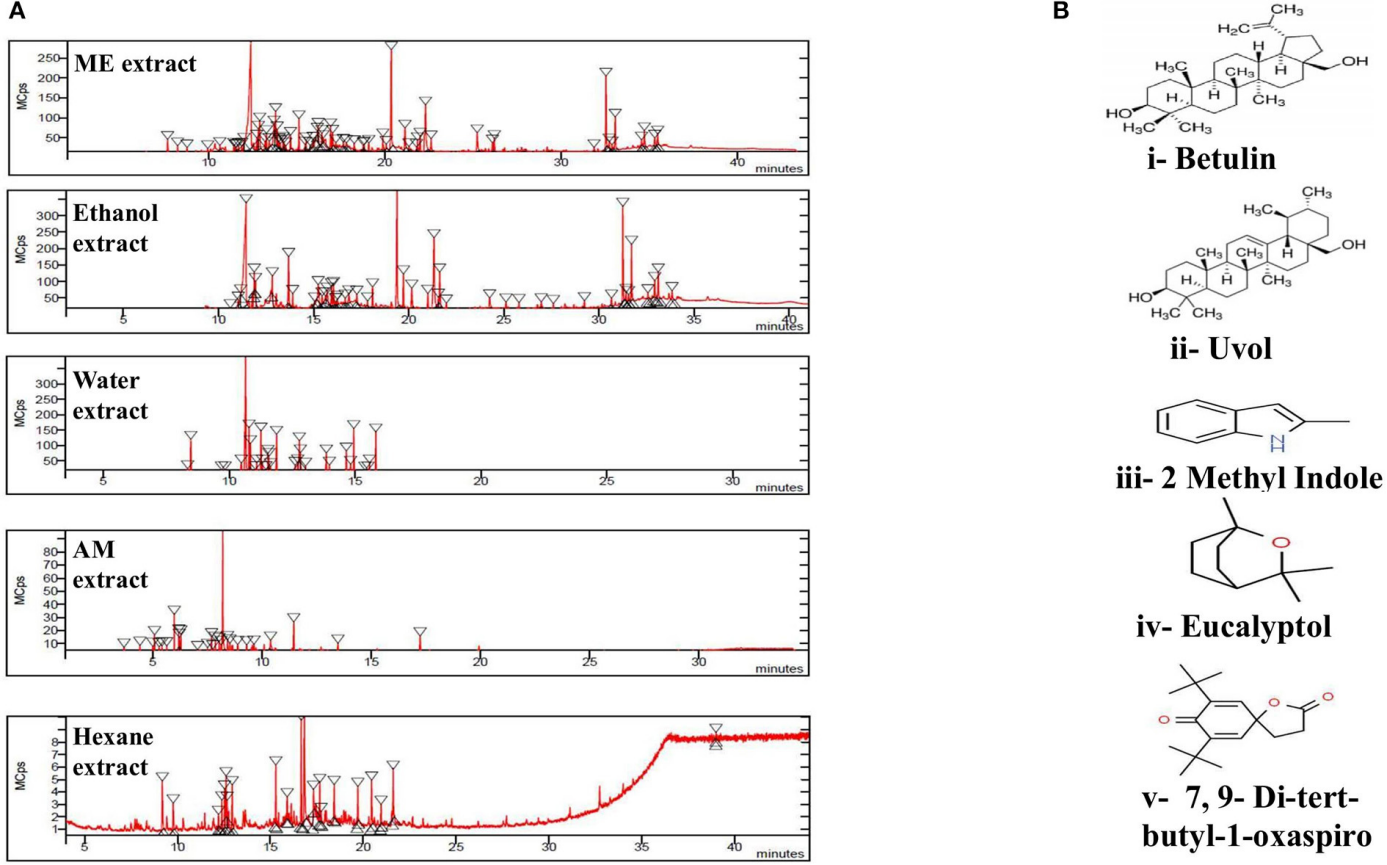
**(A)** GC/MS chromatogram of set of extracts of *Nepeta cataria* showing peaks of metabolites in each extract. **(B)** The 2-D structures of important phytochemicals are retrieved via PubChem. i. Betulin was most abundant phytochemical in methanol and ethanol (ME), ii. Uvol in ethanol, iii. 2-methyl Indole in water, iv. Eucalyptol in acetone and methanol (AM) and v. 7,9-Di-ter-butyl-1-oxaspiro is most abundant phytochemical in hexane extract.

The GC/MS analysis of a methanolic extract of *N. cataria* showed (68 identified phytochemicals + 48 unmatched) chemicals (Table 2). Analysis of ethanol-based extracts confirmed the existence of 79 known phytochemical constituents, while 31 unmatched chemicals were detected (Table 3). Water-based extracts of *N. cataria* contain 28 known phytochemicals, while 11 unmatched chemicals were also detected (Table 4). Acetone-based extract confirmed the existence of 13 known compounds' extract, while 9 chemical constituents were unmatched (Table 5). Analysis of hexane-based extracts confirmed the presence of 9 known chemical constituents, while 8 unmatched chemicals were detected, as given in Table 6. GC/MS spectral chromatograms of all the solvent-based extracts are given in Figure 5 along with the most abundant metabolite in each extract. In methanol, water, and acetone extract, 1-isopropylcyclohex-1-ene was the most abundant phytochemical. The most abundant metabolite in ethanol extract is 9,12,15-octadecatrienoic acid, and the most abundant phytochemical in hexane extract is 7,9-di-tert-butyl-1-oxaspiro (Figure 5).

**Table 2:** GC/MS analysis of a methanol extract of *N. cataria* using NIST 17 Library showed (68 identified phytochemicals + 48 unmatched) chemicals, arranged according to concentration present.

**Compound**	**Mol. formula**	**Amount/Conc.%**	**Mol. weight (g/mol)**	**RT** **(Min)**	**Extract**
1-Isopropylcyclohex-1-ene	C_9_H_16_	27.376	124.22	12.402	Methanol
Bicyclo [2.2.1] heptan-2-one,	C_7_H_10_O	20.437	110.15	7.728	Methanol
gamma. -Sitosterol	C_29_H_50_O	8.626	414.7	33.566	Methanol
Eucalyptol	C_10_H_18_O	8.505	154.249	5.112	Methanol
n-Hexadecanoic acid	C_16_H_32_O_2_	7.973	256.4241	20.364	Methanol
No match	–	6.419	–	6.933	Methanol
9,12,15-Octadecatrienoic acid	C_18_H_30_O_2_	6.401	278.43	22.304	Methanol
1-Isopropylcyclohex-1-ene	C_9_H_16_	6.144	124.22	13.699	Methanol
1,6-Octadien-3-ol, 3,7-dimet	C_10_H_18_O	5.855	154.25	9.981	Methanol
Ethyl 2-5-methyl-5-vinyltet	C_13_H_22_O_4_	5.845	242.3114	6.551	Methanol
Beta-Sitosterol	C_29_H_50_O	5.461	414.71	32.541	Methanol
No match	–	4.148	–	13.303	Methanol
No match	–	3.893	–	22.205	Methanol
Pentane, 1-chloro-5- methyl	C_5_H_11_Cl	3.739	106.594	10.696	Methanol
No match	–	3.063	–	12.903	Methanol
No match	–	3.008	–	13.718	Methanol
Bicyclo [3.1.0] hexane-2-undec	C_6_H_10_	2.974	82.14	13.804	Methanol
No match	–	2.786	–	26.376	Methanol
Alpha-Amyrin	C_30_H_50_O	2.691	426.729	33.062	Methanol
Pregnan-18-ol, 20-methyl-20-	C_22_H_39_NO	2.64	333.6	13.916	Methanol
No match	–	2.619	–	11.726	Methanol
No match	–	2.43	–	14.296	Methanol
No match	–	2.074	–	21.141	Methanol
No match	–	2.021	–	14.919	Methanol
No match	–	1.975	–	25.235	Methanol
Caryophyllene oxide	C_15_H_24_O	1.916	220.35	15.129	Methanol
No match	–	1.807	–	11.016	Methanol
No match	–	1.659	–	34.964	Methanol
No match	–	1.498	–	16.925	Methanol
No match	–	1.447	–	14.094	Methanol
No match	–	1.436	–	27.233	Methanol
No match	–	1.43	–	35.912	Methanol
2H-1-Benzopyran-2-one, 7-met	C_13_H_15_NO_2_	1.381	217.26	18.071	Methanol
Uvaol	C_30_H_50_O_2_	1.365	442.7	36.319	Methanol
No match	–	1.326	–	35.143	Methanol
Trans-Z-alpha-Bisabolene	C15H24	1.312	204.35	16.216	Methanol
Ursolic aldehyde	C_30_H_48_O_2_	1.302	440.7	34.718	Methanol
No match	–	1.279	–	7.678	Methanol
No match	–	1.245	–	17.965	Methanol
Methyl 8,11,14-heptadecatrie	C_21_H_36_O_2_	1.22	320.5093	22.864	Methanol
No match	–	1.179	–	12.826	Methanol
Phytol	C_20_H_40_O	1.179	128.1705	21.998	Methanol
No match	–	1.148	–	13.285	Methanol
No match	–	1.08	–	13.897	Methanol
No match	–	1.013	–	26.209	Methanol
No match	–	0.997	–	35.231	Methanol
Octadecanoic acid	C_18_H_36_O_2_	0.97	284.48	22.623	Methanol
Hexadecanoic acid, methyl es	C_17_H_34_O_2_	0.954	270.5	19.887	Methanol
No match	–	0.937	–	12.007	Methanol
Methyl 8,11,14-heptadecatrie	C_21_H_36_O_2_	0.92	320.5093	21.853	Methanol
Betulin	C_30_H_50_O_2_	0.91	442.72	35.472	Methanol
1,1,4a-Trimethyl-5,6-dimethyl	C_15_H_24_	0.891	204.35	33.896	Methanol
Coumarin	C_9_H_6_O_2_	0.878	146.1427	13.867	Methanol
No match	–	0.875	–	12.736	Methanol
2H-1-Benzopyran-2-one, 7-met	C_13_H_15_NO_2_	0.826	217.26	17.04	Methanol
1-Chlorosulfonyl-3-methyl-1-	C_9_H_14_ClNO_3_S	0.823	251.73	16.173	Methanol
Beta-Amyrin	C_30_H_50_O	0.763	426.729	33.739	Methanol
Methyl 2-hydroxy-octadeca-9,	C_19_H_32_O_3_	0.754	308.5	28.775	Methanol
Hexadecanoic acid, 2-hydroxy	C_16_H_32_O_3_	0.744	272.42	26.101	Methanol
No match	–	0.717	–	13.206	Methanol
(1R,7S, E)-7-Isopropyl-4,10-d	C_15_H_24_O	0.702	220.3505	17.243	Methanol
No match	–	0.688	–	35.27	Methanol
Campesterol	C_28_H_48_O	0.657	400.68	32.877	Methanol
Urs-12-en-28-al	C_30_H_48_O	0.654	424.7	35.305	Methanol
2-Butyl-5-methyl-3-2-methyl	C_15_H_26_O	0.645	222.37	14.281	Methanol
Caryophylla-4(12),8(13)-dien	C_15_H_24_O	0.632	220.3505	16.429	Methanol
endo-Borneol	C_10_H_18_O	0.623	154.25	8.246	Methanol
1-Methyl-2-methylenecyclohex	C_8_H_14_	0.622	110.197	14.461	Methanol
No match	–	0.616	–	27.717	Methanol
Caryophylla-4(12),8(13)-dien	C_15_H_24_O	0.603	220.3505	17.45	Methanol
Stigmasterol	C_29_H_48_O	0.595	412.69	33.091	Methanol
No match	–	0.585	–	14.134	Methanol
No match	–	0.579	–	13.446	Methanol
Tritetracontane	C_43_H_88_	0.574	605.2	27.798	Methanol
No match	–	0.566	–	15.531	Methanol
(3S,3aS,6R,7R,9aS)-1,1,7-Tri	C_15_H_24_	0.562	204.3511	19.087	Methanol
Megastigmatrienone	C_13_H_18_O	0.56	190.28	16.78	Methanol
No match	–	0.553	–	12.88	Methanol
No match	–	0.549	–	11.886	Methanol
Urs-12-en-28-oic acid, 3-hyd	C_30_H_48_O_3_	0.546	456.7	35.636	Methanol
No match	–	0.545	–	22.421	Methanol
No match	–	0.543	–	12.559	Methanol
3,5-Dimethylcyclohex-1-ene-4	C_8_H_14_	0.542	110.2	14.226	Methanol
Eicosanoic acid	C_20_H_40_O_2_	0.515	312.5304	25.775	Methanol
No match	–	0.486	–	13.019	Methanol
Olean-12-en-3-ol, acetate,	C_32_H_52_O_2_	0.486	468.8	32.724	Methanol
Alpha-Tocospiro A	C_29_H_50_O_4_	0.484	462.7	30.208	Methanol
Cyclohexene,1-propyl-	C_9_H_16_	0.483	124.22	11.611	Methanol
Alpha-Tocospiro B	C_29_H_50_O_4_	0.463	462.7049	30.023	Methanol
No match	–	0.447	–	11.436	Methanol
No match	–	0.447	–	12.434	Methanol
Phenol, 2,4-bis 1-methyl-1-p	C_24_H_26_O	0.445	330.5	26.725	Methanol
11,11-Dimethyl-4,8-dimethyl	C_15_H_24_O	0.429	220.35	16.954	Methanol
No match	–	0.419	–	26.522	Methanol
Tricyclo [20.8.0.07,16] tria	C30H52O2	0.413	444.7	18.261	Methanol
No match	–	0.394	–	17.818	Methanol
1,5,7-Octatrien-3-ol, 3,7-di	C_10_H_16_O	0.39	152.2334	8.782	Methanol
2-Pentadecanone, 6,10,14-tri	C_18_H_36_O	0.386	268.4778	18.826	Methanol
11,14-Octadecadienoic acid	C_18_H_32_O_2_	0.364	280.4	22.811	Methanol
No match	–	0.363	–	15.481	Methanol
Caryophylla-4(12),8(13)-dien	C_15_H_24_O	0.358	220.3505	15.937	Methanol
No match	–	0.356	–	34.665	Methanol
5-Cholestene-3-ol, 24-methyl	C_28_H_48_O	0.344	400.7	31.863	Methanol
No match	–	0.325	–	14.381	Methanol
No match	–	0.323	–	11.703	Methanol
No match	–	0.322	–	21.365	Methanol
Neophytadiene	C_20_H_38_	0.313	278.5	18.782	Methanol
No match	–	0.304	–	17.223	Methanol
2-Furanmethanol, 5-ethenylte	C_10_H_18_O_2_	0.287	170.2487	6.078	Methanol
9,12-Hexadecadienoic acid, m	C_16_H_28_O_2_	0.273	252.39	21.796	Methanol
Beta-Guaiene	C_15_H_24_	0.271	204.351	32.882	Methanol
6-Hydroxy-4,4,7a-trimethyl-5	C_11_H_16_O_3_	0.258	196.24	17.648	Methanol
Bicyclo [2.2.1] heptane, 7,7-d	C_9_H_16_	0.24	124.22	9.955	Methanol
2-Cyclohexen-1-one, 3-methyl	C_7_H_10_O	0.23	110.15	11.529	Methanol
Hentriacontane	C_31_H_64_	0.188	436.85	28.969	Methanol
Methyl octadec-6,9-dien-12-y	C_18_H_32_O_2_	0.149	280.4	15.763	Methanol

**Table 3:** GC/MS analysis of ethanol extract of *N. cataria* using NIST 17 Library showed (79 identified phytochemicals + 31 unmatched) chemicals, arranged according to concentration present.

**Compound**	**Mol. formula**	**Amount/Conc.%**	**Mol. weight** **(g/mol)**	**RT** **(Min)**	**Extract**
No match	–	57.084	–	2.058	Ethanol
No match	–	42.916	–	2.039	Ethanol
9,12,15-Octadecatrienoic acid	C_18_H_30_O_2_	27.308	278.43	17.266	Ethanol
1-Isopropylcyclohex-1-ene	C_9_H_16_	25.854	124.22	11.456	Ethanol
1-Isopropylcyclohex-1-ene	C_9_H_16_	14.94	124.22	9.585	Ethanol
1-Isopropylcyclohex-1-ene	C_9_H_16_	13.741	124.22	9.33	Ethanol
Beta-Sitosterol	C_29_H_50_O	13.312	414.71	24.939	Ethanol
n-Hexadecanoic acid	C_16_H_32_O_2_	10.3	256.424	19.386	Ethanol
Alpha-Amyrin	C_30_H_50_O	6.667	426.729	25.504	Ethanol
No match	–	4.606	–	16.278	Ethanol
Urs-12-en-28-ol	C_30_H_50_O	4.295	426.7	23.833	Ethanol
Methyl 13,14-octadecadienoate	C_19_H_34_O_2_	3.793	294.472	13.689	Ethanol
Octadecanoic acid	C_18_H_36_O_2_	3.62	284.48	17.464	Ethanol
Hexadecanoic acid, ethyl est	C_18_H_36_O_2_	3.361	284.477	15.865	Ethanol
Ethyl 9,12,15-octadecatrieno	C_20_H_34_O_2_	3.315	306.5	21.626	Ethanol
Phytol	C_20_H_40_O	3.068	128.1705	16.907	Ethanol
Coumarin	C_9_H_6_O_2_	2.94	146.1427	9.646	Ethanol
1-Chlorosulfonyl-3-methyl-1-	C_9_H_14_ClNO_3_S	2.175	251.73	15.242	Ethanol
Ursolic aldehyde	C_30_H_48_O_2_	2.109	440.7	33.113	Ethanol
Ethyl 9.cis., 11.trans.-octad	C_20_H_38_O_2_	2.045	310.515	17.352	Ethanol
No match	–	1.95	–	11.165	Ethanol
No match	–	1.756	–	15.716	Ethanol
No match	–	1.66	–	9.685	Ethanol
4,4,8-Trimethyltricyclo [6.3].	C_15_H_26_O_2_	1.458	238.366	18.101	Ethanol
No match	–	1.456	–	15.943	Ethanol
2H-1-Benzopyran-2-one, 7-met	C_13_H_15_NO_2_	1.381	217.26	16.049	Ethanol
No match	–	1.199	–	9.727	Ethanol
Hentriacontane	C_31_H_64_	1.197	436.85	20.74	Ethanol
Tetracontane, 3,5,24-trimeth	C_43_H_88_	1.194	605.2	20.201	Ethanol
6-Octadecynoic acid, methyl	C_19_H_36_O_2_	1.149	296.488	24.253	Ethanol
Eicosanoic acid	C_20_H_40_O_2_	1.138	312.5304	19.118	Ethanol
Sulfurous acid, butyl tetrad	C_21_H_44_O_3_S	1.134	376.6	23.243	Ethanol
Uvaol	C_30_H_50_O_2_	1.125	442.7	24.513	Ethanol
Bicyclo [3.1.0] hexane-2-undec	C_6_H_10_	1.108	82.14	12.837	Ethanol
Tetracosamethyl-cyclododecas	C_16_H_32_	1	224.425	27.703	Ethanol
No match	–	0.984	–	12.821	Ethanol
Octadecanoic acid, 17-methyl	C_20_H_40_O_2_	0.982	312.5	17.68	Ethanol
No match	–	0.939	–	12.875	Ethanol
Methyl 2-hydroxy-octadeca-9,	C_19_H_32_O_3_	0.895	308.5	21.548	Ethanol
2H-1-Benzopyran-2-one, 7-met	C_13_H_15_NO_2_	0.893	217.26	12.956	Ethanol
No match	–	0.884	–	11.045	Ethanol
No match	–	0.865	–	13.906	Ethanol
No match	–	0.836	–	15.628	Ethanol
[1,1′-Bicyclopropyl]-2-octan	C_21_H_38_O_2_	0.823	322.5	16.857	Ethanol
11,14-Octadecadienoic acid,	C_18_H_32_O_2_	0.819	280.4	21.561	Ethanol
Betulin	C_30_H_50_O_2_	0.802	442.72	33.839	Ethanol
No match	–	0.772	–	19.585	Ethanol
5-Hydroxymethylfurfural	C_6_H_6_O_3_	0.768	126.11	6.985	Ethanol
No match	–	0.754	–	12.601	Ethanol
No match	–	0.742	–	11.881	Ethanol
No match	–	0.727	–	12.675	Ethanol
Urs-12-en-28-oic acid, 3-hyd	C_30_H_48_O_3_	0.722	456.7	23.776	Ethanol
Sulfurous acid, butyl tetrad	C_21_H_44_O_3_S	0.667	376.6	22.185	Ethanol
No match	–	0.665	–	11.947	Ethanol
Alpha-Tocospiro A	C_29_H_50_O_4_	0.654	462.7	22.498	Ethanol
Oleic Acid	C_18_H_34_O_2_	0.653	282.47	16.515	Ethanol
Tricyclo [20.8.0.07,16] tria	C_18_H_24_O_4_	0.647	304.38	25.158	Ethanol
No match	–	0.643	–	11.217	Ethanol
Stigmasterol	C_29_H_48_O	0.63	412.69	24.507	Ethanol
Methyl 10,11-tetradecadienoa	C_15_H_26_O_2_	0.573	238.366	10.069	Ethanol
Sulfurous acid, butyl tridec	C_17_H_36_O_3_S	0.572	320.5	22.897	Ethanol
No match	–	0.562	–	12.242	Ethanol
24-Noroleana-3,12-diene	C_29_H_46_	0.537	394.676	31.418	Ethanol
No match	–	0.534	–	9.47	Ethanol
No match	–	0.517	–	22.775	Ethanol
Cholestan-3-ol, 2-methylene-	C_28_H_48_O	0.515	400.7	15.446	Ethanol
Tetracontane, 3,5,24-trimeth	C_43_H_88_	0.506	605.2	25.112	Ethanol
No match	–	0.501	–	26.914	Ethanol
2-Methylindoline	C_9_H_11_N	0.49	133.19	6.58	Ethanol
No match	–	0.481	–	16.473	Ethanol
No match	–	0.457	–	8.924	Ethanol
3,7,11,15-Tetramethyl-2-Hexa	C_20_H_40_O	0.444	296.5	23.191	Ethanol
No match	–	0.435	–	10.634	Ethanol
1-Heptatriacotanol	C_37_H_76_O	0.432	537	13.943	Ethanol
No match	–	0.424	–	13.117	Ethanol
1R,4S,7S,11R-2,2,4,8-Tetrame	C_15_H_26_O	0.419	222.366	31.553	Ethanol
No match	–	0.406	–	10.249	Ethanol
Sulfurous acid, butyl tridec	C_17_H_36_O_3_S	0.399	320.5	24.233	Ethanol
No match	–	0.395	–	16.248	Ethanol
No match	–	0.375	–	25.618	Ethanol
No match	–	0.369	–	23.972	Ethanol
6-Hydroxy-4,4,7a-trimethyl-5	C_11_H_16_O_3_	0.367	196.24	16.663	Ethanol
Ethyl 9.cis.,11. trans.-octad	C_20_H_38_O_2_	0.34	310.515	17.345	Ethanol
Tau-Cadinol	C_15_H_26_O	0.335	222.37	12.143	Ethanol
24(H)-Benzofuranone, 5,6,7,7	C_11_H_16_O_2_	0.319	180.244	10.757	Ethanol
Glycine, N-[3alpha, 5beta]	C_30_H_53_NO_4_Si	0.313	519.8	24.109	Ethanol
Tetracontane, 3,5,24-trimeth	C_43_H_88_	0.304	605.2	20.193	Ethanol
2-Pentadecanone, 6,10,14-tri	C_18_H_36_O	0.298	268.478	17.839	Ethanol
Neophytadiene	C_20_H_38_	0.294	278.5	25.337	Ethanol
2-Pentadecanone, 6,10,14-tri	C_18_H_36_O	0.286	268.478	14.346	Ethanol
2,4-Dihydroxy-2,5-dimethyl-3	C_6_H_8_O_4_	0.284	144.12	3.404	Ethanol
n-Propyl 9,12-hexadecadienoa	C_19_H_34_O_2_	0.262	294.5	11.116	Ethanol
Tetradecanoic acid	C_14_H_28_O_2_	0.25	228.3709	13.552	Ethanol
10,10-Dimethyl-2,6-dimethyle	C_15_H_24_	0.199	204.351	12.067	Ethanol
Ergost-5-en-3-ol (3beta)-	C_28_H_48_O	0.18	400.7	24.141	Ethanol
Fumaric acid, ethyl 2-methyl	C_10_H_14_O_4_	0.179	198.22	11.356	Ethanol
Tritetracontane	C_43_H_88_	0.177	605.2	22.18	Ethanol
Azulene, 1,2,3,3a,4,5,6,7-oc	C_15_H_24_	0.17	204.351	15.056	Ethanol
(4aS,7S,7aR)-4,7-Dimethyl-2	C_10_H_14_O_2_	0.169	166.217	10.885	Ethanol
cis-5,8,11,14,17-Eicosapenta	C_20_H_30_O_2_	0.148	302.5	13.276	Ethanol
Carbamic acid, N-[1,1-bis tr]	C_12_H_24_N_2_O_4_	0.124	260.33	13.319	Ethanol
Bicyclo [4.4.0] dec-1-ene, 2-i	C_15_H_24_	0.116	204.35	11.54	Ethanol
2-Cyclohexen-1-one, 4,5-dime	C_8_H_12_O	0.113	124.18	10.585	Ethanol
12-Methyl-E, E-2,13-octadecad	C_19_H_36_O	0.113	280.489	11.164	Ethanol
Stigmasterol	C_29_H_48_O	0.102	412.69	24.241	Ethanol
2-Cyclohexen-1-one, 3-methyl	C_7_H_10_O	0.083	110.15	8.592	Ethanol
Megastigmatrienone	C_13_H_18_O	0.081	190.28	11.924	Ethanol
2,4-Dihydroxy-2,5-dimethyl-3	C_6_H_8_O_4_	0.032	144.12	3.23	Ethanol
Cyclopentanecarboxylic acid	C_6_H_10_O_2_	0.008	114.14	9.434	Ethanol
2-Methylindoline	C_9_H_11_N		133.19	8.12	Ethanol

**Table 4:** GC/MS analysis of water extract of *N. cataria* using NIST 17 Library showed (79 identified phytochemicals + 31 unmatched) chemicals, arranged according to concentration present.

**Compound**	**Mol. formula**	**Amount/Conc.%**	**Mol. weight (g/mol)**	**RT (Min)**	**Extract**
1-Isopropylcyclohex-1-ene	C_9_H_16_	22.387	124.22	10.657	Water
7-Methylhexahydrocyclopenta	C_9_H_14_O_2_	5.399	154.21	11.265	Water
2H-1-Benzopyran-2-one, 7-met	C_13_H_15_NO_2_	5.336	217.26	14.95	Water
(R) -(-)-14-Methyl-8-hexadecy	C_17_H_34_O	5.106	254.4513	10.79	Water
No match	–	4.917	–	15.825	Water
Benzofuran, 2,3-dihydro-	C_8_H_8_O	4.002	120.15	8.48	Water
Hydro coumarin	C_9_H_8_O_2_	3.699	148.1586	10.843	Water
Bicyclo [3.1.0] hexane-2-undec	C_6_H_10_	3.1	82.14	12.831	Water
No match	–	2.942	–	13.861	Water
Coumarin	C_9_H_6_O_2_	2.265	146.1427	11.545	Water
Cyclopentane carboxylic acid,	C_6_H_10_O_2_	2.165	114.14	10.486	Water
13-Tetradece-11-yn-1-ol	C_14_H_24_O	2.146	208.34	11.581	Water
No match	–	1.738	–	11.098	Water
No match	–	1.63	–	14.825	Water
No match	–	1.472	–	15.575	Water
(S-2-1R,4R)-4-Methyl-2-oxo	C_4_H_6_O_3_	1.274	102.0886	12.723	Water
(4R,4aR,7S,7aR)-4,7-Dimethyl	C_10_H_18_O	1.17	154.25	11.326	Water
Homovanillyl alcohol	C_9_H_12_O_3_	1.118	168.19	12.621	Water
2-Cyclohexene-1-one, 4-3-hyd	C_13_H_20_O_2_	0.997	208.2967	13.984	Water
No match	–	0.932	–	13.036	Water
2-Methylindoline	C_9_H_11_N	0.825	133.19	8.353	Water
(E)-2,6-Dimethylocta-3,7-die	C_10_H_18_O_2_	0.67	170.25	8.078	Water
No match	–	0.562	–	11.045	Water
Ethanone, 1-2-hydroxyphenyl	C_8_H_8_O_2_	0.559	136.15	11.463	Water
2-Methoxy-4-vinyl phenol		0.53		9.845	Water
6-Hydroxy-4,4,7a-trimethyl-5	C_11_H_16_O_3_	0.496	196.24	15.394	Water
No match	–	0.469	–	3.652	Water
No match	–	0.437	–	5.652	Water
No match	–	0.404	–	16.262	Water
3-Acetylthymine	C_5_H_6_N_2_O_2_	0.402	126.1133	13.283	Water
3-Oxo-4-phenylbutyronitrile	C_10_H_9_NO	0.371	159.18	8.825	Water
No match	–	0.337	–	4.368	Water
7-Oxabicyclo [4.1.0] heptan-3-	C_6_H_10_O_2_	0.295	114.14	16.821	Water
n-Hexadecanoic acid	C_16_H_32_O_2_	0.263	256.4241	17.288	Water
1H-Pyrrole-2,5-dione, 3-ethy	–	0.25	–	8.69	Water
1,7-Octadiene-3,6-diol, 2,6-	C_10_H_18_O_2_	0.238	170.25	9.271	Water
Conhydrin	C_8_H_17_NO	0.212	143.23	7.847	Water
Methyl 7,8-octadecadienoate	C_19_H_34_O_2_	0.206	294.4721	12.898	Water
1H-Indene, 1-ethylideneoctah	C_11_H_10_	0.07	142.2	14.737	Water

**Table 5:** GC/MS analysis of an acetone-based extract of *N. cataria* using NIST 17 Library showed (12 identified phytochemicals + 9 unmatched) chemicals, arranged according to concentration present.

**Compound**	**Mol. formula**	**Amount/conc. %**	**Mol. weight (g/mol)**	**RT (Min)**	**Extract**
Oxime-, methoxy-phenyl-	C_8_H_9_NO_2_	2.849	151.16	3.685	Acetone
1-Isopropylcyclohex-1-ene	C_9_H_16_	29.552	124.22	8.206	Acetone
Caryophyllene oxide	C_15_H_24_O	6.868	220.35	11.452	Acetone
(+)-2-Bornanone	C_10_H_16_O	6.365	152.233	5.984	Acetone
n-Hexadecanoic acid	C_16_H_32_O_2_	5.337	256.424	17.237	Acetone
No match	–	3.443	-	10.391	Acetone
Endo-Borneol	C_10_H_18_O	3.083	154.25	6.191	Acetone
Hotrienol	C_10_H_16_O	2.947	152.23	7.692	Acetone
No match	–	2.573	-	13.475	Acetone
(E)-2,6-Dimethylocta-3,7-die	C_10_H_18_O_2_	2.572	170.25	6.217	Acetone
No match	–	2.57	-	8.885	Acetone
Cyclopentanecarboxylic acid	C_6_H_10_O_2_	2.496	114.14	8.04	Acetone
No match	–	2.289	-	9.631	Acetone
No match	–	2.038	-	8.424	Acetone
No match	–	2.007	-	8.53	Acetone
No match	–	1.947	-	8.127	Acetone
No match	–	1.844	-	9.297	Acetone
Eucalyptol	C_10_H_18_O	1.513	154.249	5.004	Acetone
No match	–	1.196	-	7.749	Acetone
Cyclohexane, 1-propyl-	C_9_H_16_	1.093	124.22	7.507	Acetone
alpha-methyl- alpha-[4-methyl]	C_6_H_11_NO_2_	1.026	129.16	5.292	Acetone
1,7-Octadiene-3,6-diol, 2,6-dimethyl	C_10_H_18_O_2_	0.819	170.25	7.049	Acetone

**Table 6:** GC/MS analysis of a hexane-based extract of *N. cataria* using NIST 17 Library showed (9 identified phytochemicals + 8 unmatched) chemicals, arranged according to concentration present.

**Compound**	**Mol. formula**	**Amount/Conc. %**	**Mol. weight** **(g/mol)**	**RT** **(Min)**	**Extract**
(+)-2-Bornanone	C_10_H_16_O	6.809	152.233	9.187	Hexane
Methyl 6,9,12,15,18-heneicos	–	11.008	–	16.663	Hexane
1,2-Benzenedicarboxylic acid	C_8_H_6_O_4_	8.551	166.14	10.181	Hexane
Dibutyl phthalate	C_16_H_22_O_4_	5.877	278.34	21.611	Hexane
No match	–	5.199	–	12.947	Hexane
No match	–	4.075	–	12.537	Hexane
No match	–	3.969	–	19.713	Hexane
endo-Borneol	C_10_H_18_O	3.719	154.25	9.774	Hexane
Benzophenone	C_13_H_10_O	3.591	182.217	17.321	Hexane
No match	–	3.472	–	18.437	Hexane
No match	–	3.172	–	12.675	Hexane
Tetracontane, 3,5,24-trimeth	C_43_H_88_	2.939	605.2	8.975	Hexane
No match	–	2.756	–	12.391	Hexane
Benzoic acid, 4-ethoxy-, eth	C_11_H_14_O_3_	2.535	194.23	15.905	Hexane
7,9-Di-tert-butyl-1-oxaspiro	C_17_H_24_O_3_	1.956	276.4	20.957	Hexane
No match	–	1.421	–	12.21	Hexane
No match	–	1.176	–	17.73	Hexane

## Discussion

One of the most well-known species in the genus *Nepeta* is *N. cataria*. Several studies have performed qualitative identification of phytochemical constituents from leaves and flowers of *N. cataria* extract as well as oils from the plant (Edewor and Usman, 2011; Reichert et al., 2018; Azizian et al., 2021). The antibacterial of *N. cataria* from previous research likewise demonstrated sufficient antibacterial activity against *S. aureus, K. pneumoniae* and *S. typhi* (Mukhtar and Singh, 2019). The results from our studies corroborate the results exhibited in previous studies. In addition to *N. cataria*, other species of the *Nepeta* genus have also been studied extensively for their phytochemical analysis, and among all species, *N. cataria* is the most promising of all species (Azizian et al., 2021).

Several studies corroborate our findings and indicate high DPPH activity in acetone extracts while others exhibit versatile results (Dienaite et al., 2018). Some studies presented more efficient DPPH activity in methanol, 70% ethanol and others in aqueous extract of *N. cataria* (Kraujalis et al., 2011; Mihaylova et al., 2013; Dienaite et al., 2018). Modernized extraction protocols, i.e., ultrasound-based microextraction, are being used to maximize output of phenolic compounds from methanol extract of *N. cataria*, which corroborates with our study (Hajmohammadi et al., 2021). Several other studies also indicate rosmarinic acid as a prominent phenolic compound in *N. cataria* extracts (Hadi et al., 2017).

Water extracts of *N. cataria* exhibit reasonable ORAC activity as per different studies (Dienaite et al., 2018; Baranauskiene et al., 2019). Another study showed excellent radical scavenging properties of *N. cataria via* FRAP assay, which improves the confidence in this plant (Duda et al., 2015).

Among all the treatments, ethanol-based extracts of *N. cataria* showed maximum percentage inhibition of all the tested bacteria at 1,000–250 μg/ml concentration, followed by methanolic extracts at 1,000 and 500 μg/ml dose levels and water-based extracts at 1,000 and 500 μg/ml dose levels. In contrast, acetone and hexane-based extracts of *N. cataria* did not significantly inhibit all the tested bacterial isolates compared to control treatments. Many studies provide insights for the use of *N. cataria* extract in inhibition of *S. aureus* and *B. subtilis* and its oil as a topical treatment of respiratory tract infections (Suschke et al., 2007; Bandh and Kamili, 2011). MIC values indicated that the ethanol-based extract of all *N. cataria* extracts showed maximum inhibition of *B. subtilis*, followed by *C. freundii* and *M. luteus*. At the same time, methanol-based extracts also showed maximum efficacy against *S. sonnei, E. coli, M. luteus*, and *C. freundii*. Water, acetone, and hexane-based extracts were almost equally effective against tested bacterial isolates, as given in Table 7. Studies indicate promising effect of *N. cataria* extract as antibacterial agent against *S. aureus, K. pneumoniae*, and *Salmonella typhi* (Edewor and Usman, 2011). Considering resazurin methodology, by using combined extractions of all solvents in DMSO, *N. cataria* plant extract at the dose level of 12.5 μl/ml showed maximum inhibition of all the bacterial strains, followed by 6.25 μl/ml. The antibacterial screening of the *N. cataria* from other studies also exhibited sufficient evidence of antibacterial activity against *S. aureus, K. pneumoniae*, and *S. typhi* (Morombaye et al., 2018).

**Table 7:** Antimicrobial efficacy of *N. cataria* extracts against a set of gram-negative and gram-positive bacterial strains.

**Bacterial pathogens**		**Zone of inhibition (mm)**					
		**Methanol**	**Ethanol**	**Water**	**Acetone**	**Hexane**	**Chloramphenicol**
Gram negative	*E. coli*	15 ± 0.1	14 ± 0.1	12 ± 0.1	0	14 ± 0.1	25 ± 0.2
	*K. oxytoca*	14 ± 0.2	14 ± 0.1	16 ± 0.1	14 ± 0.2	13 ± 0.3	26 ± 0.1
	*S. enterica*	13 ± 0.1	14 ± 0.1	0	0	0	25 ± 0.1
	*S. sonnei*	15 ± 0.2	15 ± 0.1	0	16 ± 0.2	14 ± 0.1	26 ± 0.2
	*C. ferundii*	15 ± 0.2	22 ± 0.4	12 ± 0.1	12 ± 0.2	11 ± 0.1	25 ± 0.2
Gram positive	*B. subtilis*	14 ± 0.1	21 ± 0.5	0	0	14 ± 0.2	31 ± 0.1
	*L. lactis*	0	0	0	13 ± 0.1	0	25 ± 0.2
	*L. monocytogenes*	13 ± 0.1	14 ± 0.2	13 ± 0.1	13 ± 0.1	0	25 ± 0.2
	*M. luteus*	15 ± 0.2	16 ± 0.2	16 ± 0.1	16 ± 0.2	0	26 ± 0.1
	*S. aureus*	13 ± 0.1	13 ± 0.1	0	0	0	20 ± 0.1

GC/MS analysis of methanol and ethanol revealed the presence of betulin extracts, which is a promising antitumorigenic candidate and escalates the importance of *N. cataria* in cancer treatment (Liu et al., 2009). Arachidic acid (eicosanoic acid) is used to produce detergents, photographic materials, and lubricants. Caryophyllene oxide is a potential preservative used in food, drugs, and cosmetics. It also displays anti-inflammatory and anti-carcinogenic properties (Salaria et al., 2020). Uvaol also displays anti-inflammatory properties and antioxidant effects (Botelho et al., 2019). Campesterols found in methanol extracts is phytosterol, used in growth induction in animals, commonly abused anabolic steroid in sports can also reduce the absorption of cholesterol in intestine by targeting transporter protein, minimizing the effect of cardiovascular disease (Choudhary and Tran, 2011). Phytol in ethanol has been investigated for its potential anxiolytic, metabolism-modulating, cytotoxic, antioxidant, autophagy- and apoptosis-inducing, antinociceptive, anti-inflammatory, immune-modulating, and antimicrobial effects (Islam et al., 2018). Phytol is likely the most abundant acyclic isoprenoid compound present in the biosphere and its degradation products have been used as biogeochemical tracers in aquatic environments (Rontani and Volkman, 2003). Phytol is used in the fragrance industry and is used in cosmetics, shampoos, toilet soaps, household cleaners, and detergents (McGinty et al., 2010). Coumarin (2*H*-1-benzopyran-2-one) in methanol and ethanol is famous for pharmacological properties such as anti-inflammatory, anticoagulant, antibacterial, antifungal, antiviral, anticancer, antihypertensive, antitubercular, anticonvulsant, antiadipogenic, antihyperglycemic, antioxidant, and neuroprotective properties (Venugopala et al., 2013). Similarly in water extracts, 2-methylindole is used as an intermediate to synthesize dyes, pigments, and pharmaceuticals. Conhydrin is a poisonous alkaloid, when ingested interruption with the central nervous system, paralyzing respiratory muscles and causing failure (Hotti and Rischer, 2017). Likewise, extracts of hexane contain eucalyptol, an active ingredient as a cough suppressant as it controls mucus secretion from airway and asthma *via* anti-inflammatory cytokines (Juergens, 2014). Hexane soluble constituents conformed to identification of 7, 9-di-tert-butyl-1-oxaspiro which is used against skin diseases, gonorrhea, migraine, intestinal parasites, and warts (Sharif et al., 2015), and dibutyl phthalate is used in making flexible plastics. In addition to this, several other studies indicate presence of nepetalactone and other terpenoids as essential components of oil extracts of *N. cataria* (Handjieva et al., 2011; Sharma et al., 2019).

This study gave a thorough brief of antibacterial and antioxidant activity and its constituents. Present methodology can be beneficial in devising and exploring different bioactive compounds that can be exploited for the constructing novel antimicrobial agents for alternative therapeutic intervention against several bacterial and viral infections after processing. It may also help to treat different antibiotic-resistant pathogens. Its chemicals if used in pharmacology industries can serve as indigenous, cheaper, and readily available source.

## Conclusion

Many aspects of plants were studied, but complete metabolomic profiling and identification of unmatched chemicals remain a question mark. MS-MS analysis of plant metabolites should be considered for knowing the medicinal potential of unknown and novel plant metabolites. Data compilation and individual chemical studies need a larger scale with a set of skills to combat emerging diseases. Yet, to the best of our knowledge, the concluded information, reported results, and this research is comprehensive to the best of our scale, our team tried to achieve.

## Data availability statement

The original contributions presented in the study are included in the article/supplementary material, further inquiries can be directed to the corresponding author.

## Author contributions

Practical performance and data compilation were performed solely by AN. Experimental assistance for GC/MS, and antibacterial analysis was given by BA. Data analysis was performed by HS. Manuscript drafting and proofreading were conducted by HS, in assistance with MW and AT. All authors contributed to the study design and implementation. All authors contributed to the article and approved the submitted version.

## Funding

The authors acknowledge the Higher Education Commission, Government of Pakistan, for funding part of the research under the International Research Support Initiative Program (IRSIP) at the University of Massachusetts, USA.

## Conflict of interest

The authors declare that the research was conducted in the absence of any commercial or financial relationships that could be construed as a potential conflict of interest.

## Publisher's note

All claims expressed in this article are solely those of the authors and do not necessarily represent those of their affiliated organizations, or those of the publisher, the editors and the reviewers. Any product that may be evaluated in this article, or claim that may be made by its manufacturer, is not guaranteed or endorsed by the publisher.

## References

[B1] Adiguzel A. Ozer H. Sokmen M. Gulluce M. Sokmen A. Kilic H.. (2009). Antimicrobial and antioxidant activity of the essential oil and methanol extract of *Nepeta cataria*. Polish J. Microbiol. 58, 69-76. Available at: www.microbiology.pl/pjm 19469289

[B2] Angelini P. Pagiotti R. Menghini A. Vianello B. (2006). Antimicrobial activities of various essential oils against foodborne pathogenic or spoilage moulds. Ann. Microbiol. 56, 65–69. 10.1007/BF03174972

[B3] Azizian T. Alirezalu A. Hassani A. Bahadori S. Sonboli A. (2021). Phytochemical analysis of selected Nepeta species by HPLC-ESI-MS/MS and GC-MS methods and exploring their antioxidant and antifungal potentials. J. Food Meas. Charact. 15, 2417–2429. 10.1007/s11694-021-00819-8

[B4] Bandh S. Kamili A. (2011). Evaluation of antimicrobial activity of aqueous extracts of *Nepeta cataria* Cellular and Humoral Immune Responses following Immunization with Surface/excretory secretory Antigens of Abomasal Nematodes of Sheep View project BOOK PROJECTS View project. Artic. J. Pharm. Res. Available online at: https://www.researchgate.net/publication/235769154 (accessed July 14, 2022).

[B5] Baranauskiene R. BendŽiuviene V. RagaŽinskiene O. Venskutonis P. R. (2019). Essential oil composition of five Nepeta species cultivated in Lithuania and evaluation of their bioactivities, toxicity and antioxidant potential of hydrodistillation residues. Food Chem. Toxicol. 129, 269–280. 10.1016/j.fct.2019.04.039 31029727

[B6] Botelho R. M. Tenorio L. P. G. Silva A. L. M. Tanabe E. L. L. Pires K. S. N. Gonçalves C. M.. (2019). Biomechanical and functional properties of trophoblast cells exposed to Group B Streptococcus *in vitro* and the beneficial effects of uvaol treatment. Biochim. Biophys. Acta Gen. Subj. 1863, 1417–1428. 10.1016/j.bbagen.2019.06.012 31254547

[B7] Bourrel C. Perineau F. Michel G. Bessiere J. M. (2011). Catnip (*Nepeta cataria* L.) essential oil: analysis of chemical constituents, bacteriostatic and fungistatic properties. J. Essent. Oil Res. 5, 159–167. 10.1080/10412905.1993.9698195

[B8] Choudhary S. Tran L. (2011). Phytosterols: perspectives in human nutrition and clinical therapy. Curr. Med. Chem. 18, 4557–4567. 10.2174/092986711797287593 21864283

[B9] Dabiri M. Sefidkon F. (2003). Chemical composition of *Nepeta crassifolia* Boiss. and Buhse oil from Iran. Flavour Fragr. J. 18, 225–227. 10.1002/ffj.1199

[B10] Dienaite L. Pukalskiene M. Matias A. A. Pereira C. V. Pukalskas A. Venskutonis P. R. (2018). Valorization of six Nepeta species by assessing the antioxidant potential, phytochemical composition and bioactivity of their extracts in cell cultures. J. Funct. Foods 45, 512–522. 10.1016/j.jff.2018.04.004

[B11] Duda S. C. Mărghita,ş L. A. Dezmirean D. Duda M. Mărgăoan R. Bobi,ş O. (2015). Changes in major bioactive compounds with antioxidant activity of *Agastache foeniculum, Lavandula angustifolia, Melissa officinalis* and *Nepeta cataria*: effect of harvest time and plant species. Ind. Crops Prod. 77, 499–507. 10.1016/j.indcrop.2015.09.045

[B12] Edewor I. T. Usman A. L. (2011). Phytochemical and antibacterial activities of leaf extracts of *Nepeta cataria*. African J. Pure Appl. Chem. 5, 503–506. 10.5897/AJPAC11.074

[B13] Elshikh M. Ahmed S. Funston S. Dunlop P. McGaw M. Marchant R.. (2016). Resazurin-based 96-well plate microdilution method for the determination of minimum inhibitory concentration of biosurfactants. Biotechnol. Lett. 38, 1015–1019. 10.1007/s10529-016-2079-2 26969604 PMC4853446

[B14] Ergün Z. (2021). Seed oil content and fatty acid profiles of endemic *Phoenix theophrasti* greuter, *Phoenix roebelenii* o'brien, *Phoenix caneriensis* hort. Ex chabaud, and *Phoenix dactylifera* l. grown in the same locality in Turkey. Turkish J. Agric. For. 45, 557–564. 10.3906/tar-2105-34

[B15] Frolova N. Uktainets A. Korablova O. Voitsekhivskyi V. (2020). Plants of *Nepeta cataria* var. citriodora Beck. and essential oils from them for food industry. Potravin. J. Food Sci. 13, 449–455. 10.5219/1109

[B16] Giarratana F. Muscolino D. Ziino G. Lo Presti V. Rao R. Chiofalo V.. (2017). Activity of Catmint (*Nepeta cataria*) essential oil against Anisakis larvae. Trop. Biomed. 34, 22–31. 33592976

[B17] Gilani A. H. Shah A. J. Zubair A. Khalid S. Kiani J. Ahmed A.. (2009). Chemical composition and mechanisms underlying the spasmolytic and bronchodilatory properties of the essential oil of *Nepeta cataria* L. J. Ethnopharmacol. 121, 405–411. 10.1016/j.jep.2008.11.004 19041706

[B18] Hadi N. Sefidkon F. Shojaeiyan A. Šiler B. Jafari A. A. Aničić N.. (2017). Phenolics' composition in four endemic Nepeta species from Iran cultivated under experimental field conditions: the possibility of the exploitation of *Nepeta germplasm. Ind. Crops Prod*. 95, 475–484. 10.1016/j.indcrop.2016.10.059

[B19] Hajmohammadi M. R. Najafi AsliPashaki S. Rajab Dizavandi Z. Amiri A. (2021). Ultrasound-assisted vesicle-based microextraction as a novel method for determination of phenolic acid compounds in *Nepeta cataria* L. samples. J. Iran. Chem. Soc. 18, 1559–1566. 10.1007/s13738-020-02131-6

[B20] Handjieva N. V. Popov S. S. Evstatieva L. N. (2011). Constituents of essential oils from *Nepeta cataria* L., *N. grandiflora* M.B. and *N. nuda* L. 8, 639–643. 10.1080/10412905.1996.9701032

[B21] Hinkov A. Angelova P. Marchev A. Hodzhev Y. Tsvetkov V. Dragolova D.. (2020). *Nepeta nuda* ssp. nuda L. water extract: inhibition of replication of some strains of human alpha herpes virus (genus simplex virus) in vitro, mode of action and NMR-based metabolomics. J. Herb. Med. 21, 100334. 10.1016/j.hermed.2020.100334

[B22] Hotti H. Rischer H. (2017). The killer of Socrates: coniine and related alkaloids in the plant kingdom. Molecule 22, 1962. 10.3390/molecules22111962 29135964 PMC6150177

[B23] Humphries R. M. Ambler J. Mitchell S. L. Castanheira M. Dingle T. Hindler J. A.. (2018). CLSI methods development and standardization working group best practices for evaluation of antimicrobial susceptibility tests. J. Clin. Microbiol. 56, e01934-17. 10.1128/JCM.01934-17 29367292 PMC5869819

[B24] Islam M. T. Ali E. S. Uddin S. J. Shaw S. Islam M. A. Ahmed M. I.. (2018). Phytol: a review of biomedical activities. Food Chem. Toxicol. 121, 82–94. 10.1016/j.fct.2018.08.032 30130593

[B25] Juergens U. R. (2014). Anti-inflammatory properties of the monoterpene 18-cineole: current evidence for co-medication in inflammatory airway diseases. Drug Res. (Stuttg). 64, 638–646. 10.1055/s-0034-1372609 24831245

[B26] Kaewprom K. Chen Y. H. Lin C. F. Chiou M. T. Lin C. N. (2017). Antiviral activity of *Thymus vulgaris* and *Nepeta cataria* hydrosols against porcine reproductive and respiratory syndrome virus. Thai J. Vet. Med. 47, 25–33.

[B27] Khan T. Khan M. A. Mashwani Z. ur R. Ullah N. Nadhman A. (2021). Therapeutic potential of medicinal plants against COVID-19: the role of antiviral medicinal metabolites. Biocatal. Agric. Biotechnol. 31, 101890. 10.1016/j.bcab.2020.101890 33520034 PMC7831775

[B28] Kraujalis P. Rimantas Venskutonis P. Ragazinskiene O. (2011). “Antioxidant activities and phenolic composition of extracts from nepeta plant species,” in Proceedings of the 6th Baltic Conference on Food Science and Technology.36015417

[B29] Liu H. Wang S. Cai B. Yao X. (2009). Anticancer activity of compounds isolated from *Engelhardtia serrata* Stem Bark. Pharm. Biol. 42, 475–477. 10.3109/13880200490889028

[B30] Lucas-Abellán C. Mercader-Ros M. T. Zafrilla M. P. Fortea M. I. Gabaldón J. A. Núñez-Delicado E. (2008). ORAC-fluorescein assay to determine the oxygen radical absorbance capacity of resveratrol complexed in cyclodextrins. J. Agric. Food Chem. 56, 2254–2259. 10.1021/jf0731088 18303815

[B31] Mamadalieva N. Z. Akramov D. K. Ovidi E. Tiezzi A. Nahar L. Azimova S. S.. (2017). Aromatic medicinal plants of the Lamiaceae family from Uzbekistan: ethnopharmacology, essential oils composition, and biological activities. Medicine 4, 8. 10.3390/medicines4010008 28930224 PMC5597069

[B32] McGinty D. Letizia C. S. Api A. M. (2010). Fragrance material review on phytol. Food Chem. Toxicol. 48, S59–S63. 10.1016/j.fct.2009.11.012 20141879

[B33] Mihaylova D. Popova A. Deseva I. N. (2013). In vitro antioxidant activity and phenolic composition of *Nepeta cataria* L. extracts. Int. J. Agric. Sci. Technol. 1, 74–79.

[B34] Mir M. A. Parihar K. Tabasum U. Kumari E. Mir A. Amin Mir M. (2016). Estimation of alkaloid, saponin and flavonoid, content in various extracts of *Crocus sativa*. J. Med. Plants Stud. 4, 171–174.

[B35] Morombaye S. M. Kangogo M. Revathi G. Nyerere A. Ochora J. Morombaye S. M.. (2018). Evaluation of the antimicrobial effect of *Nepeta cataria* and *Basella alba* against clinically resistant *Acinetobacter baumannii* in Nairobi, Kenya. Adv. Microbiol. 8, 790–803. 10.4236/aim.2018.810052

[B36] Mukhtar H. M. Singh G. P. (2019). Pharmacognostic and phytochemical investigations of aerial parts of *Nepeta cataria* Linn. Asian J. Pharm. Pharmacol. 5, 810–815. 10.31024/ajpp.2019.5.4.23

[B37] Nadeem A. Ahmed B. Shahzad H. Craker L. E. Muntean T. (2021). *Verbascum Thapsus* (Mullein) versatile polarity extracts: GC-MS analysis, phytochemical profiling, anti-bacterial potential and anti-oxidant activity. Res. Artic. Pharmacogn. J. 13, 1488–1497. 10.5530/pj.2021.13.189

[B38] Naguib A. M. M. Ebrahim M. E. Aly H. F. Metawaa H. M. Mahmoud A. H. Mahmoud E. A.. (2012). Phytochemical screening of *Nepeta cataria* extracts and their in vitro inhibitory effects on free radicals and carbohydrate-metabolising enzymes. Nat. Product Res. 26, 2196–2198. 10.1080/14786419.2011.635342 22103287

[B39] Narimani R. Moghaddam M. Ghasemi Pirbalouti A. Mojarab S. (2017). Essential oil composition of seven populations belonging to two Nepeta species from Northwestern Iran. Int. J. Food Proper. 20, 2272–2279. 10.1080/10942912.2017.1369104

[B40] Özkan K. Karadag A. Sagdiç O. (2021). Determination of the in vitro bioaccessibility of phenolic compounds and antioxidant capacity of Juniper berry (*Juniperus drupacea* Labill.) pekmez. Turk. J. Agric. For. 45, 290–300. 10.3906/tar-2009-2

[B41] Packialakshmi N. Naziya S. (2014). Phytochemical and antimicrobial screening of the polar and non-polar solvent stem extract of *Caralluma fimbriyata*. Int. J. Pure Appl. Biosci. 2, 32–37. 10.3126/ijasbt.v2i3.10796

[B42] Pargaien A. V. Bisht N. Joshi H. Pargaien S. Adhikari M. (2020). Analysis of ethanomedicinally potential extract of *Nepeta cataria*. Eco. Env. Cons. 26, 90–95. Available online at: http://www.envirobiotechjournals.com/EEC/Vol26OctSuppl20/EEC-15.pdf21843624

[B43] Prabhavathi R. M. Prasad M. P. Jayaramu M. (2016). Studies on qualitative and quantitative phytochemical analysis of *Cissus quadrangularis*. Adv. Appl. Sci. Res. 7, 11–17.

[B44] Reichert W. Ejercito J. Guda T. Dong X. Wu Q. Ray A.. (2019). Repellency assessment of *Nepeta cataria* essential oils and isolated nepetalactones on *Aedes aegypti*. Sci. Rep. 9, 1–9. 10.1038/s41598-018-36814-1 30728370 PMC6365536

[B45] Reichert W. Villani T. Pan M. H. Ho C. T. Simon J. E. Wu Q. (2018). Phytochemical analysis and anti-inflammatory activity of *Nepeta cataria* accessions. J. Med. Active Plants 7, 19–27. 10.7275/1mca-ez51

[B46] Rontani J. F. Volkman J. K. (2003). Phytol degradation products as biogeochemical tracers in aquatic environments. Org. Geochem. 34, 1–35. 10.1016/S0146-6380(02)00185-7

[B47] Salaria D. Rolta R. Sharma N. Dev K. Sourirajan A. Kumar V. (2020). In silico and in vitro evaluation of the anti-inflammatory and antioxidant potential of *Cymbopogon citratus* from North-western Himalayas. bioRxiv 2020.05.31.124982. 10.1101/2020.05.31.124982

[B48] Sarin R. V. Bafna P. A. (2012). Herbal antidiarrhoeals: a review. Int. J. Res Pharmacol. Biomed. Sci. 3, 637–649.

[B49] Scott H. (2003). Catnip, *Nepeta cataria*, a morphological comparison of mutant and wild type specimens to gain an ethnobotanical perspective. Econ. Bot. 57, 135–142. 10.1663/0013-0001(2003)057[0135:CNCAMC]2.0.CO;2

[B50] Sharif H. B. Mukhtar M. D. Mustapha Y. Lawal A. O. (2015). Preliminary investigation of bioactive compounds and bioautographic studies of whole plant extract of *Euphorbia pulcherrima* on *Escherichia coli, Staphylococcus aureus, Salmonella typhi*, and *Pseudomonas aeruginosa*. Adv. Pharm. 2015, 14. 10.1155/2015/485469

[B51] Sharma A. Cooper R. Bhardwaj G. Cannoo D. S. (2021). The genus Nepeta: traditional uses, phytochemicals and pharmacological properties. J. Ethnopharmacol. 268, 113679. 10.1016/j.jep.2020.113679 33307050

[B52] Sharma A. Nayik G. A. Cannoo D. S. (2019). Pharmacology and Toxicology of Nepeta cataria (Catmint) Species of Genus Nepeta: A Review. Cham: Springer.

[B53] Suschke U. Sporer F. Schneele J. Geiss H. K. Reichling J. (2007). Antibacterial and cytotoxic activity of *Nepeta cataria* L., *N. cataria* Var. Citriodora (Beck.) Balb. and *Melissa officinalis* L. essential oils. Nat. Prod.Commun. 2, 1277–1286. 10.1177/1934578X0700201218

[B54] Tan J. Li J. Ma J. Qiao F. (2019). Hepatoprotective effect of essential oils of *Nepeta cataria* L. On acetaminophen-induced liver dysfunction. Biosci. Rep. 39, BSR20190697. 10.1042/BSR20190697 31337687 PMC6684950

[B55] Venugopala K. N. Rashmi V. Odhav B. (2013). Review on natural coumarin lead compounds for their pharmacological activity. Biomed Res. Int. 2013, 963248. 10.1155/2013/963248 23586066 PMC3622347

[B56] Yang F. Chen Y. Xue Z. Lv Y. Shen L. Li K.. (2020). High-throughput sequencing and exploration of the lncRNA-circRNA-miRNA-mRNA network in type 2 diabetes Mellitus. Biomed. Res. Int. 2020, 13. 10.1155/2020/8162524 32596376 PMC7273392

